# CD44-mediated hyaluronan binding marks proliferating hematopoietic progenitor cells and promotes bone marrow engraftment

**DOI:** 10.1371/journal.pone.0196011

**Published:** 2018-04-23

**Authors:** Sally S. M. Lee-Sayer, Meghan N. Dougan, Jesse Cooper, Leslie Sanderson, Manisha Dosanjh, Christopher A. Maxwell, Pauline Johnson

**Affiliations:** 1 Department of Microbiology and Immunology, University of British Columbia, Vancouver, B.C., Canada; 2 Department of Pediatrics, British Columbia Children’s Hospital Research Institute, University of British Columbia, Vancouver, B.C., Canada; Emory University, UNITED STATES

## Abstract

CD44 is a widely expressed cell adhesion molecule that binds to the extracellular matrix component, hyaluronan. However, this interaction is not constitutive in most immune cells at steady state, as the ability of CD44 to engage hyaluronan is highly regulated. While activated T cells and macrophages gain the ability to bind hyaluronan by CD44, the status in other immune cells is less studied. Here we found a percentage of murine eosinophils, natural killer and natural killer T cells were capable of interacting with hyaluronan at steady state. To further investigate the consequences of hyaluronan binding by CD44 in the hematopoietic system, point mutations of CD44 that either cannot bind hyaluronan (LOF-CD44) or have an increased affinity for hyaluronan (GOF-CD44) were expressed in CD44-deficient bone marrow. Competitive bone marrow reconstitution of irradiated mice revealed an early preference for GOF-CD44 over WT-CD44 expressing cells, and for WT-CD44 over LOF-CD44 expressing cells, in the hematopoietic progenitor cell compartment. The advantage of the hyaluronan-binding cells was observed in the hematopoietic stem and progenitor populations, and was maintained throughout the immune system. Hematopoietic stem cells bound minimal hyaluronan at steady state, and this was increased when the cells were induced to proliferate whereas multipotent progenitors had an increased ability to bind hyaluronan at steady state. *In vitro*, the addition of hyaluronan promoted their proliferation. Thus, proliferating hematopoietic progenitors bind hyaluronan, and hyaluronan binding cells have a striking competitive advantage in bone marrow engraftment.

## Introduction

The cell surface glycoprotein CD44 is ubiquitously expressed by mammalian cells and its interaction with its ligand, hyaluronan (HA), a component of the extracellular matrix, is tightly regulated by hematopoietic cells [1]. At steady state, most immune cells do not bind significant levels of fluorescently labeled HA (FL-HA), indicating a low likelihood of engagement with endogenous HA, which is present in most tissues [2]. Alveolar macrophages are an exception [3–5], where their engagement with HA promotes their survival in the alveolar space [6]. In other macrophages, the ability for CD44 to bind HA is induced after activation, as documented after monocyte activation [7, 8], monocyte-derived dendritic cell activation [9], and LPS and IFNγ- or IL-4-induced macrophage activation [10]. In lymphoid cells, including conventional and regulatory T cells [11–14], B cells [15, 16], and natural killer (NK) cells [17], HA binding is inducible and observed in a subset of activated cells. In general, once a threshold level has been achieved, high CD44 expression correlates with FL-HA binding [1]. The most strongly activated T cells express the highest levels of CD44 and bind HA (unpublished results), and FL-HA binding identifies the most proliferative activated T cells [11]. A transient interaction of activated T cells with HA on inflamed endothelial cells promotes rolling and extravasation at inflammatory sites [18, 19]. While the expression level of CD44 and HA binding status for these immune cell populations have been well characterized [1], characterization of other populations is lacking. Furthermore, the heterogeneous pattern of HA binding by immune populations suggests that different cell types have different requirements for interaction with HA. However despite the importance of HA in embryonic development [20], the consequences of CD44 binding HA during immune development and function are not well understood. CD44^-/-^ mice develop normally with a functioning immune system [21], suggesting that either CD44 plays a non-essential and redundant role, or these mice compensate for the loss of CD44 [22, 23]. Alternatively, HA-binding cells may out-compete non-HA binding cells for HA-rich niches in CD44^+/+^ wildtype (WT) mice, making any effect of HA binding hard to distinguish when comparing CD44^+/+^ and CD44^-/-^ mice. Here we sought to determine the HA binding potential of immune cells and evaluate the consequences of HA binding by immune cells in mice, using a loss of HA-binding CD44 mutant (LOF-CD44), generated by mutation of a critical arginine (R43 in mouse CD44) in the HA-binding site [24, 25], and a constitutive HA-binding form of CD44 (GOF-CD44), created by mutation of a serine residue (S183 in mouse CD44) that prevents the addition of inhibitory chondroitin sulfate [26]. LOF-CD44 and GOF-CD44 were expressed in CD44^-/-^ bone marrow (BM) cells (BMC) and used in competitive BM reconstitution experiments, to assess the impact of HA binding on hematopoietic cell development and function.

## Materials and methods

### Mice

C57BL/6 mice (CD45.2) and B6.SJL-Ptprca Pep3 (CD45.1) mice were obtained from Jackson Laboratories. CD45.2 and CD45.1 homozygous mice were crossed once to generate the CD45.1/2 heterozygous mice. These and CD44^-/-^ mice [21], backcrossed onto C57BL/6 for nine generations, were bred and maintained in specific pathogen-free conditions at the University of British Columbia (UBC) animal facilities. Animal experiments were performed in accordance with the protocols approved by the UBC Animal Care Committee and Canadian Council of Animal Care guidelines (A11-0316, A15-0208). Mice were euthanized by gradual exposure to carbon dioxide to minimize stress and used between 6 and 9 weeks of age at the start of experiments and were matched for sex and age for comparative studies. For systemic bacterial infection, mice received 30,000 CFU of *Listeria monocytogenes* (*Lm*) intravenously (i.v.) [11].

### Antibodies and reagents

Streptavidin or antibodies against mouse CD3 (17A2), CD4 (GK1.5), CD8α (53–6.7), CD11b (M1/70), CD11c (N418), CD16/32 (2.4G2), CD19 (1D3), CD34 (RAM34), CD44 (IM7.8), CD45-B220 (RA3 6B2), CD45.1 (A20), CD45.2 (104), CD48 (HM48-1), CD49b (DX5), CD117/c-kit (2B8), CD127 (A7R34), CD135 (A2F10), CD150 (mSHAD150), F4/80 (BM8), IgM (II/41), Ly6G/ Gr1 (RB6-8C5), MHC II (M5/114.15.2), NK1.1 (PK136), Sca-1 (D7), Siglec F (E50-2440), TCRβ (H57-597), γδTCR (eBioGL3) and Ter119 (TER-119) conjugated to biotin, PE, PE-Cy5, PE-CF594, PE-Cy5.5, PE-Cy7, PerCP-Cy5.5, allophycocyanin, Alexa Fluor® 647, Alexa Fluor® 405, Alexa Fluor® 700, or Pacific Blue were purchased from eBioscience, BD Biosciences or BRC AbLab. 4',6-diamidino-2-phenylindole (DAPI; Sigma-Aldrich) or the LIVE/DEAD® Fixable Aqua Dead Cell Stain Kit (Molecular Probes) were used to identify dead cells. 7-aminoactinomycin D (7AAD) (eBiosience) was used for cell cycle analysis. Rooster comb HA (Sigma-Aldrich) was conjugated to fluorescein according to [27] or to Alexa Fluor® 647 (BRC AbLab) and used to detect HA binding.

### Flow cytometry (FC)

Single cell suspensions were generated and labeled [11]. Briefly, peripheral (brachial, axillary and inguinal) lymph nodes (pLN), spleens and thymuses were passed through a 70 μm filter, and BMC were flushed with FC buffer (2% BSA, 2 mM EDTA in PBS) from the tibia and femur of mice using a 26 ½ gauge needle. Blood was collected from cardiac puncture of recently euthanized mice. After red blood cell lysis, cells were labeled with antibodies and FL-HA, after first incubating with an antibody against CD16/32 to block and sometimes also stain for Fc receptors. Dead cells were labeled by 25 ng/ ml DAPI or by LIVE/DEAD® Fixable Dead Cell Stain. For cell cycle analysis, cells were fixed in ice-cold 75% ethanol overnight at -20°C, washed once in PBS, incubated with 0.5 mg/ mL ribonuclease A (Pharmacia) at 37°C for 10 minutes, and then 7AAD was added to a final concentration of 0.05 mg/ mL for 30 minutes at room temperature. Labeled cells were analyzed on an LSRII or Canto flow cytometer (BD Biosciences) using FACSDiva acquisition software (BD Biosciences). Data analyses were performed using FlowJo (Tree Star).

### BM isolation and culture

BMC were isolated from tibia and femurs, and then cultured in complete Dulbecco's modified eagle medium (DMEM) (high glucose DMEM, with 15% FCS, and 50 U/ ml penicillin/ streptomycin, 1 mM sodium pyruvate, and 2 mM L-glutamine, all from Thermo Fisher Scientific) supplemented with 100 ng/ mL stem cell factor (SCF), 6 ng/ mL interleukin- (IL-)3 and 10 ng/ mL IL-6 (3/6/SCF medium; all cytokines from Cedarlane). For retroviral transduction, BMC were isolated from CD45.2 CD44^-/-^ mice injected i.v. with 150 mg/ kg of 5-fluorouracil (5-FU, Sigma-Aldrich) dissolved in 1X PBS four days prior. For *in vitro* cultures, lineage^+^ cells were depleted by labeling cells with biotinylated antibodies against CD4, CD8α, CD11b, CD11c, B220, NK1.1 Ter119. For carrier cells used in BM transfer, Sca-1^+^ cells were depleted using biotinylated antibody against Sca-1 and anti-biotin microbeads (Miltenyi Biotec), followed by removal by LS columns (Miltenyi Biotec). To add immobilized exogenous HA *in vitro*, 5 or 50 μg of high molecular weight HA (Lifecore Biomedical) dissolved in sterile milliQ water was dried onto 96-well plates in the biological safety cabinet overnight.

### Retroviral transduction and BM reconstitution

CD44 constructs with bicistronically expressed green or red fluorescent protein (GFP or RFP, respectively) reporter genes, and the retrovirus packaging cell lines from [28] were used. Packaging cell line E+GP86 was cultured in complete DMEM [29]. 5-FU treated BMC were cultured at 1–1.5 x 10^6^ cells/ mL for two days in 3/6/SCF. Cells were then co-cultured for two additional days in fresh 3/6/SCF medium with the packaging cells that had been treated with 10 μg/ mL of mitomycin C (Sigma-Aldrich) a day prior. This culture enriched for lineage^-^ and progenitor cells in the BM. Transduced cells were sorted based on CD44 expression and GFP or RFP expression on the BD Influx or BD Aria by the UBCFlow Facility. Typically, 30–45% of the cells expressed the retroviral construct.

CD45.1 BM recipient mice were irradiated with two doses of 650 rads, four hours apart. One day later, irradiated mice received 1.8–2.2 x 10^5^ of the transduced, sorted CD45.2 BMC. BMC were co-transferred with 2 x 10^5^ Sca-1-depleted carrier cells from the BM of CD45.1 mice in 3 experiments. Alternatively, BMC from CD45.1/2 CD44^+/+^ or CD45.2 CD44^-/-^ mice were isolated, and a 1:1 ratio of 8 x 10^6^ total BMC were transferred into irradiated CD45.1 host mice.

### Statistical analysis

Flow analysis was performed using FlowJo (Tree Star). Two-tailed Student’s t-tests were used to analyze the data and statistical differences were considered significant when p < 0.05.

## Results

### Immune cells exhibit different HA binding profiles at steady state

**Fig 1:**
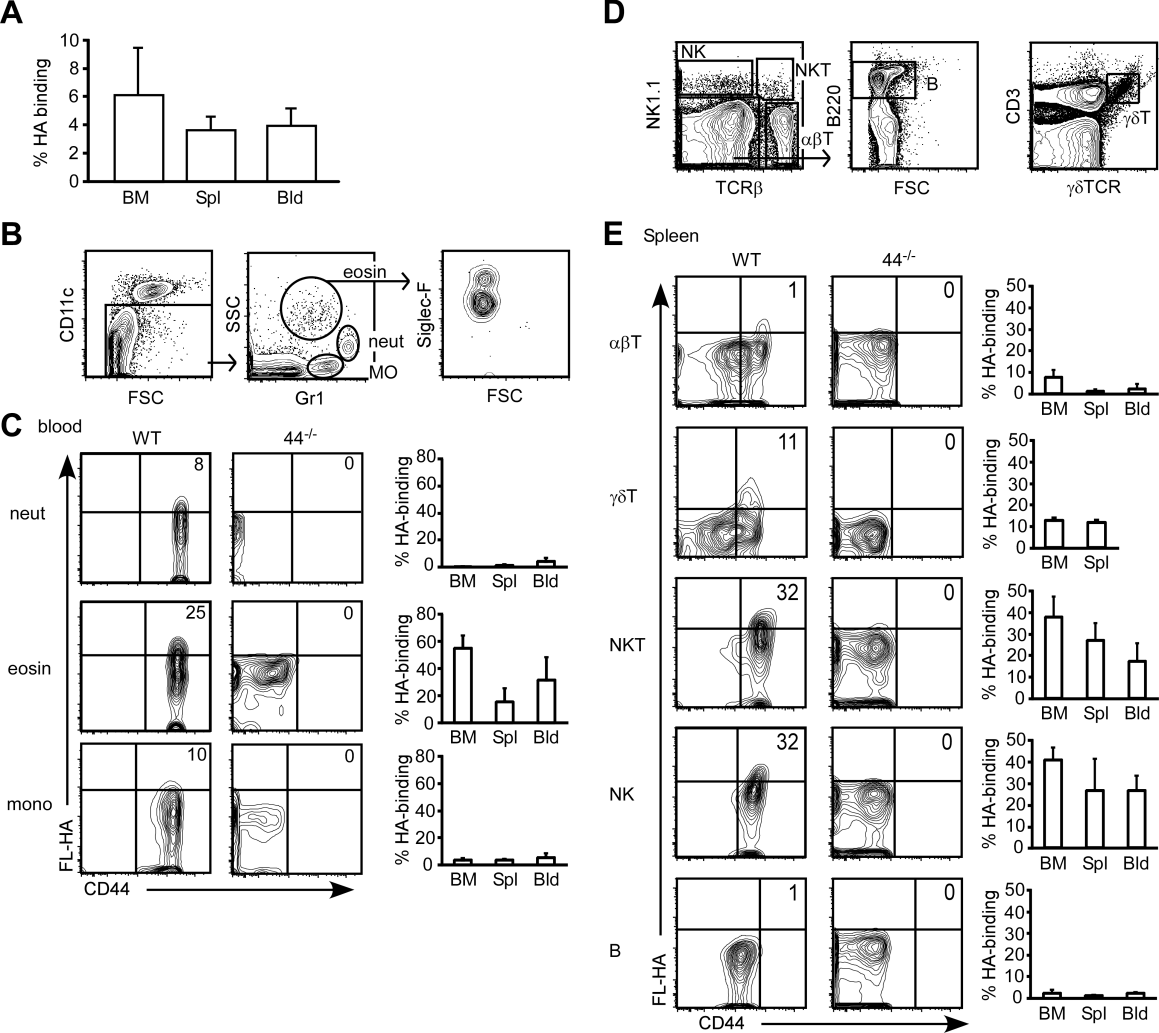
Immune cells from the BM, spleen and blood exhibit different CD44 expression and HA binding. (A) Percentage of FL-HA binding by CD44^+/+^ CD45.2 BMC, splenocytes and blood cells. (B) Gating strategies for neutrophils, eosinophils and monocytes. (C) FC plots of FL-HA binding versus CD44 expression by neutrophils, eosinophils and monocytes in CD44^+/+^ or CD44^-/-^ blood, and graphs showing average percentages of FL-HA binding in BM, spleen and blood. (D) Gating strategies for conventional αβT, γδT, NK, NKT and B cells. (E) FC plots of FL-HA binding versus CD44 expression by conventional αβT, γδT, NK, NKT and B cells in CD44^+/+^or CD44^-/-^ spleen, graphs showing average percentages of FL-HA binding in BM, spleen and blood. Mean +/- SD from six biological replicates of two independent experiments.

CD44-mediated HA binding by various immune cell populations was assessed using FL-HA and compared with cells from CD44^-/-^ mice, which do not bind HA. Less than 10% of total BMC, splenocytes and blood cells bound FL-HA (Fig 1A). Monocytes, neutrophils and eosinophils were identified based on their expression of CD11c, Gr1 and granularity (Fig 1B) and although these cells expressed high levels of CD44, only the eosinophils had a prominent FL-HA binding population (Fig 1C). T, NK, natural killer T (NKT) and B cells were identified based on their expression of NK1.1, TCRβ and B220 (Fig 1D). In agreement with published findings, αβ T cells and B cells from naïve mice expressed low levels of CD44 and less than 10% of them bound FL-HA (Fig 1E). γδ T cells also expressed low levels of CD44 and around 10% of them bound FL-HA (Fig 1E). In contrast, NK and NKT cells had higher CD44 expression, and 20–40% of NK and NKT cells bound FL-HA (Fig 1E).

### Competitive BM reconstitution between CD44^+/+^ and CD44^-/-^ BMC reveals a slight advantage for CD44^+/+^ cells in T, NK and splenic eosinophil populations

**Fig 2:**
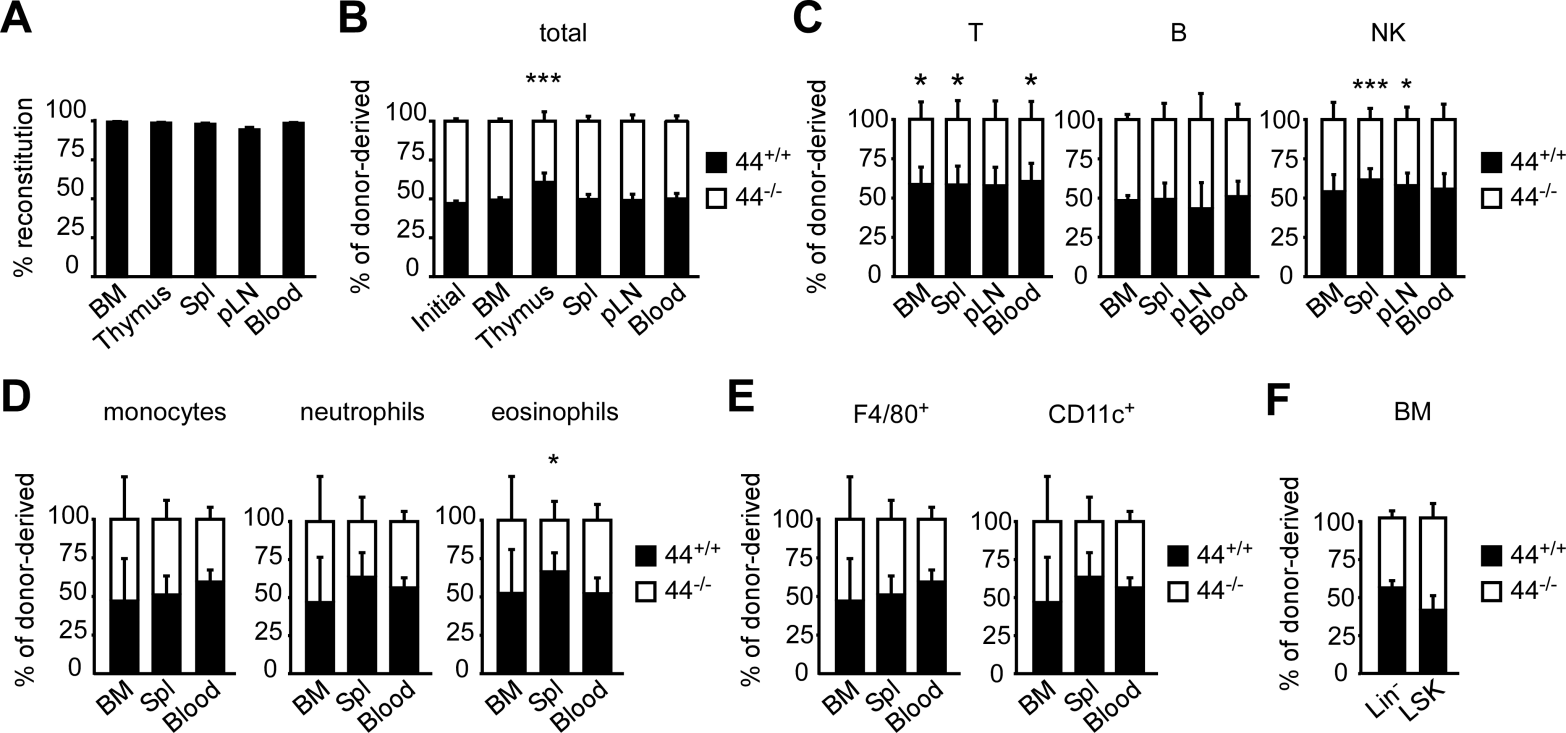
CD44^+/+^ BMC have a slight competitive advantage in reconstituting T cells, NK cells and splenic eosinophils. Lethally irradiated CD45.1 CD44^+/+^ mice were reconstituted with 8 x 10^6^ of a 1:1 mixture of CD45.1/ 45.2 CD44^+/+^ and CD45.2 CD44^-/-^ BMC for 7 weeks. (A) Reconstitution by all the CD45.2^+^ donor-derived cells. (B-F) Percentage of CD45.1/ 45.2 CD44^+/+^ and CD45.2 CD44^-/-^ cells within the donor-derived populations of total organ (B), conventional T, B and NK cell populations (C), monocyte, neutrophil and eosinophil populations (D), F4/80^+^ macrophage and CD11c^+^ DC populations (E), and lineage^-^ and LSK populations (F). Mean +/- SD from eight biological replicates of two independent experiments. *p<0.05, ***p<0.001 calculated by Student’s t-test.

Since immune cells develop normally in CD44^-/-^ mice [21], it was reasoned that any advantage conferred by CD44 and HA binding may only become apparent when CD44^+/+^ cells are in competition with CD44^-/-^ cells. Thus an equal mixture of BMC from CD45.1/2 CD44^+/+^ and CD45.2 CD44^-/-^ mice were competitively transferred into lethally irradiated CD45.1 hosts, to compare the reconstitution of different immune compartments by CD44^+/+^ and CD44^-/-^ BMC. After 7 weeks, donor-derived cells reconstituted over 95% of the BM, thymus, spleen, pLN and blood (Fig 2A). A slight but significant competitive advantage was observed for the reconstitution of thymocytes and mature T cells by CD44^+/+^ cells (Fig 2B and 2C), in line with previous reports [30]. CD44^+/+^ cells also had a competitive advantage in reconstituting NK cells in the spleen and pLN, although the differences were smaller than that for T cells (Fig 2C). No significant difference between CD44^+/+^ and CD44^-/-^ derived cells was observed for B cells, monocytes, neutrophils, F4/80^+^ and CD11c^+^ DC (Fig 2D and 2E). A slight competitive advantage for reconstitution by CD44^+/+^ cells of eosinophils was observed in the spleen (Fig 2D). CD44^**+/+**^ and CD44^-/-^ BMC were able to reconstitute the BM lineage^-^ and Lineage^-^, Sca-1^+^, c-Kit^+^ (LSK) populations similarly (Fig 2F). This shows that CD44 provides a slight competitive advantage for thymocytes, T cells, NK cells and splenic eosinophils. Since the majority of CD44^+/+^ cells do not have a high affinity for HA, this may explain why only slight differences are apparent. To further investigate the impact of HA binding by CD44, we created BMC expressing CD44 that cannot bind HA (LOF-CD44) and BMC expressing CD44 that has an increased ability to bind (GOF-CD44) [10] for competitive BM reconstitution experiments.

### GOF-CD44 expressing CD44^-/-^ BMC exhibit HA binding whereas LOF-CD44 expressing cells do not

**Fig 3:**
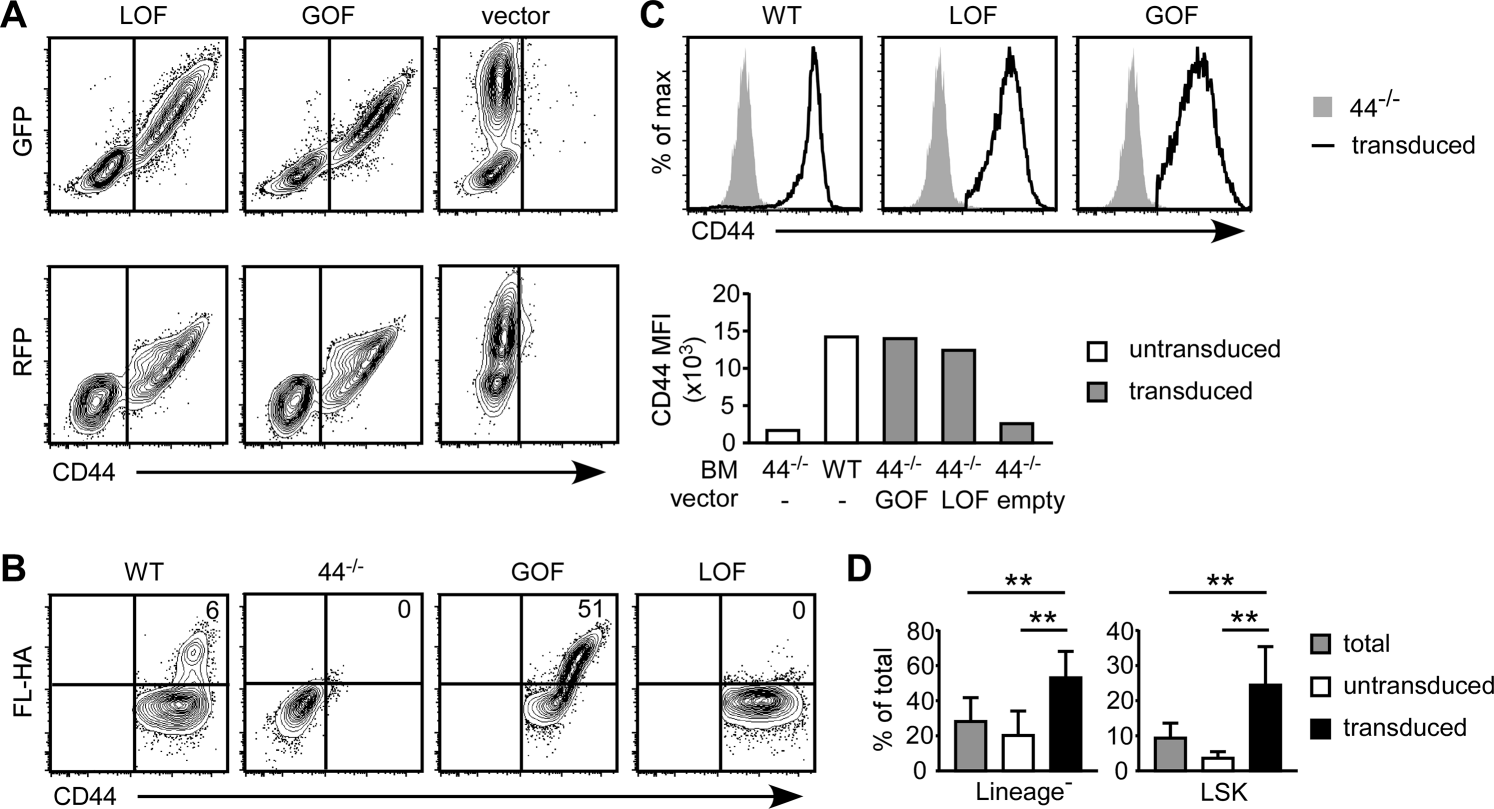
LOF-CD44 and GOF-CD44 BMC are generated using retroviral transduction. CD44^-/-^ BMC were isolated from the femurs and tibia of mice that had received 5-FU 4 days prior, and were then cultured with 3/6/SCF medium. CD44^-/-^ BMC were transduced with GOF-CD44 or LOF-CD44 constructs, and CD44^+/+^ BMC were transduced with an empty vector (WT). (A) FC plots of GFP or RFP expression versus CD44 expression. (B) FC plots of FL-HA binding versus CD44 expression of WT, CD44^-/-^, LOF-CD44 or GOF-CD44 transfected BMC. (C) Histograms and mean fluorescence intensity (MFI) of CD44 expression of WT, LOF-CD44 or GOF-CD44 expressing BMC, after gating on reporter-positive BMC. Representative of two experiments. (D) Percentage of lineage^-^ and LSK cells within total, untransduced and transduced BMC. Mean +/- SD from eight biological replicates of three independent experiments. **p<0.01 calculated by Student’s t-test.

The LOF-CD44 and GOF-CD44 constructs expressing either GFP or RFP were transduced into CD44^-/-^ BMC using retroviral infection, as described in the Methods, and GFP and RFP reporter expression correlated with CD44 expression (Fig 3A). HA binding in the transduced cells corresponded with the CD44 construct expressed. Less than 10% of CD44^+/+^ BMC transduced with the empty vector (WT-CD44) bound FL-HA, whereas LOF-CD44 cells did not bind FL-HA, and approximately 50% of GOF-CD44 cells bound HA (Fig 3B). Notably, CD44 expression levels on transduced cells were comparable to that of WT-CD44 BMC transduced with the empty vector (Fig 3C). Analysis of the transduced BMC revealed an enrichment for the lineage^-^ and LSK population, consistent with the culture of BMC in 3/6/SCF medium and the stem cell tropism of the retroviral vector (Fig 3D).

### GOF-CD44 cells dominate BM reconstitution when in competition with WT-CD44 BMC

**Fig 4:**
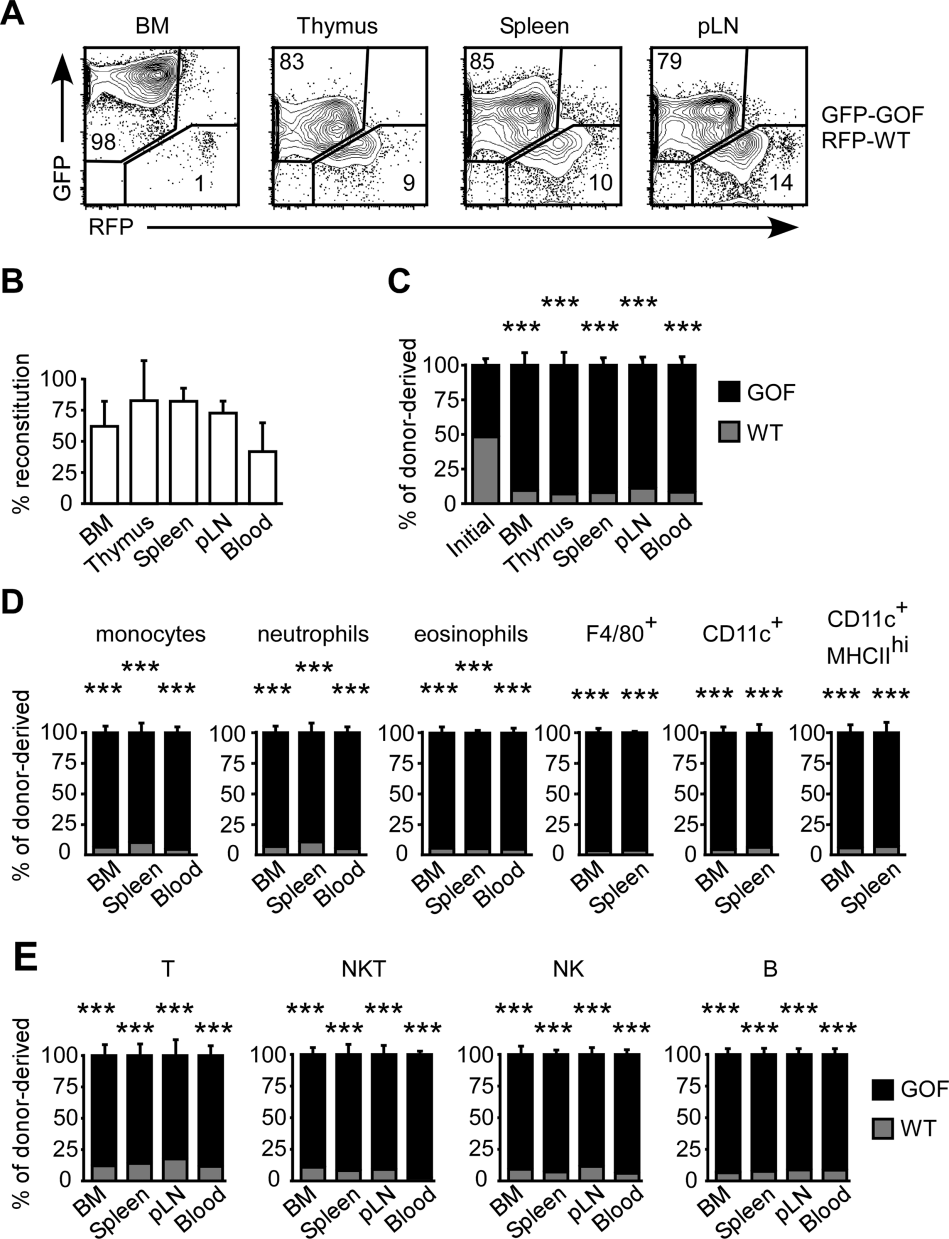
GOF-CD44 cells out-compete WT-CD44 BMC in reconstituting irradiated mice. Lethally irradiated CD45.1 mice were reconstituted with 2 x 10^5^ BMC containing an equal mix of CD45.2 RFP+ WT-CD44 and CD45.2 GFP+ GOF-CD44 for 7 or 11 weeks. (A) FC plots of GFP versus RFP expression of the CD45.2^+^ donor-derived cells. (B) Reconstitution by total CD45.2^+^ donor-derived cells. (C-E) Percentage of WT-CD44 and GOF-CD44 cells within the donor-derived total cells (C), neutrophils, eosinophils, monocytes, F4/80^+^ macrophages, total CD11c^+^ DC and CD11c^+^MHCII^hi^ mature DC populations (D), and conventional T, NKT, NK and B (B220^+^CD19^+^IgM^+^ in the BM; B220^+^ in the spleen and blood) populations. Mean +/- SD from eight biological replicates of two independent experiments. ***p<0.001 calculated by Student’s t-test.

To evaluate the effect of HA binding on the development of hematopoietic cells, an equal mixture of GFP^+^ GOF-CD44 and RFP^+^ WT-CD44 BMC were transferred into irradiated hosts. After at least 7 weeks of reconstitution, more GOF-CD44 cells were present in the BM, thymus, spleen and pLN (Fig 4A). Over 45% of the BM, thymus, spleen, pLN and blood consisted of donor-derived cells (Fig 4B), and of the donor cells, significantly lower percentages of WT-CD44 cells were found in the tissues and blood of reconstituted mice (Fig 4A and 4C). A significant competitive advantage of GOF-CD44 cells over WT-CD44 cells was observed in the reconstitution of monocytes, neutrophils and eosinophils in the BM, spleen and blood, as well as F4/80^+^, CD11c^+^ and CD11c^+^ MHCII^hi^ cells in the BM and spleen, where over 85% of the cells were GOF-CD44 (Fig 4D). Likewise, over 80% of the T, NK, NKT and B cells in the BM, spleen, pLN and blood, were GOF-CD44 (Fig 4E).

### WT-CD44 cells dominate BM reconstitution when in competition with LOF-CD44 BMC

**Fig 5:**
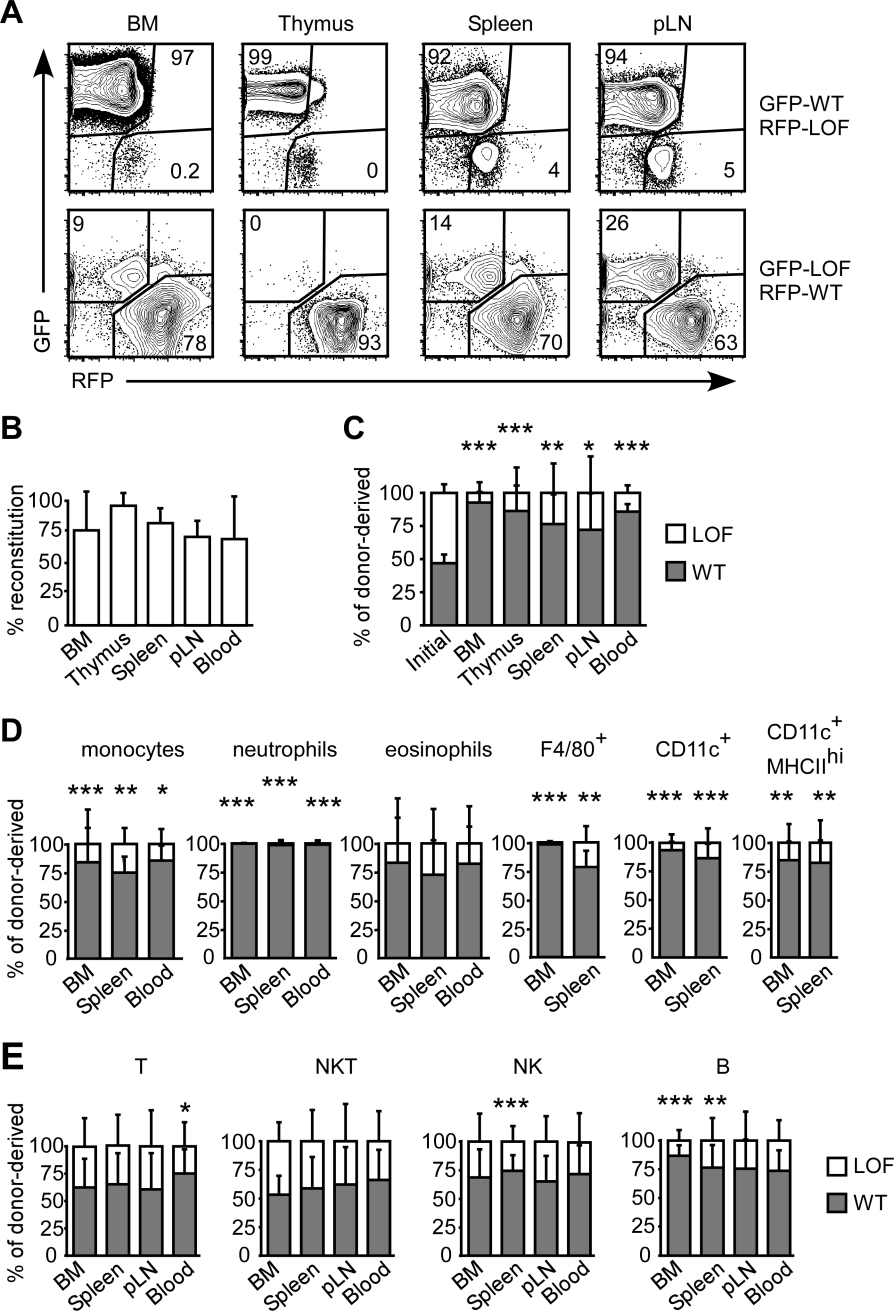
WT-CD44 cells out-compete LOF-CD44 BMC in reconstituting irradiated mice. Lethally irradiated CD45.1 mice were reconstituted with 2 x 10^5^ BMC containing an equal mix of CD45.2 WT-CD44 and LOF-CD44 for 7 or 11 weeks. (A) FC plots of GFP versus RFP expression of the CD45.2^+^ donor-derived cells. (B) Reconstitution by total CD45.2^+^ donor-derived cells. (C-E) Percentage of WT-CD44 and LOF-CD44 cells within the donor-derived total cells (C), neutrophils, eosinophils, monocytes, F4/80^+^ macrophages, total CD11c^+^ DC and CD11c^+^MHCII^hi^ mature DC populations (D), and conventional T, NKT, NK and B (B220^+^CD19^+^IgM^+^ in the BM; B220^+^ in the spleen and blood) populations. Mean +/- SD from six biological replicates of two independent experiments. *p<0.05, **p<0.01, ***p<0.001 calculated by Student’s t-test.

To evaluate the loss of HA binding on the development of hematopoietic cells, an equal mixture of LOF-CD44 and WT-CD44 expressing BMC were transferred into irradiated hosts and allowed to reconstitute for over 7 weeks. LOF-CD44 cells expressed either GFP or RFP, and WT-CD44 cells expressed the other color fluorescent protein, to determine that the results were not influenced by reporter expression. Over 65% of the BM, thymus, spleen, pLN and blood consisted of donor-derived cells, and of these cells, WT-CD44 cells reconstituted between 70–95% of the donor-derived cells in these organs, significantly out-competing the LOF-CD44 cells, irrespective of reporter expression (Fig 5A and 5C). The competitive advantage of WT-CD44 cells over LOF-CD44 cells was reflected in the reconstitution of mature myeloid populations, where WT-CD44 cells reconstituted 75–100% of the donor-derived monocytes, neutrophils and eosinophils in the BM, spleen and blood, and F4/80^+^, CD11c^+^ and CD11c^+^ MHCII^hi^ cells in the BM and spleen, and the differences were significant for all populations, except for eosinophils (Fig 5D). More WT-CD44 cells were found within the T, NK, NKT and B cell populations in the BM, spleen, pLN and blood. However, the difference was smaller and generally mostly insignificant in these populations, as WT-CD44 cells made up 55–75% of the T and NKT cell populations, and 65–85% of the NK and B cell populations (Fig 5E).

### HA binding cells provide an early advantage in BM reconstitution

**Fig 6:**
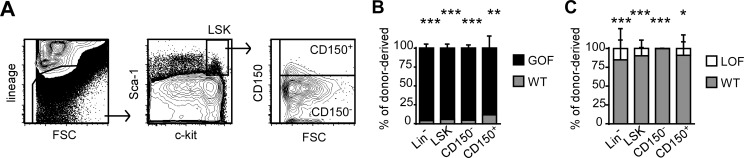
HA binding BMC confer a competitive advantage in BM progenitor reconstitution. (A) Gating strategies for Lineage^-^ BM, LSK, and CD150. (B-C) Percentage of WT-CD44 and GOF-CD44 (B) or LOF-CD44 cells (C) within the donor-derived BM lineage^-^, LSK, CD150^+^ LSK and CD150^-^ LSK populations. Mean +/- SD from at least six biological replicates of two independent experiments. *p<0.05, ***p<0.001 calculated by Student’s t-test.

The competitive advantage of GOF-CD44 cells over WT-CD44 cells, and of WT-CD44 cells over LOF-CD44 cells in most of the hematopoietic cells examined suggested that the advantage was occurring early during BM reconstitution. The LSK population was further divided into CD150^+^ and CD150^-^ populations, which contain the HSC and MPP, respectively (Fig 6A). GOF-CD44 cells made up the majority (over 85%) of BM lineage^-^ cells, as well as the total, CD150^+^ and CD150^-^ LSK populations (Fig 6B), and WT-CD44 cells significantly out-competed LOF-CD44 cells to a similar extent in the same populations (Fig 6C). These competition studies revealed an *in vivo* role for HA binding in reconstituting the BM progenitors, where the increased ability to bind HA conferred a competitive advantage to the BMC.

### Less HSC bind HA than downstream progenitors in the BM

**Fig 7:**
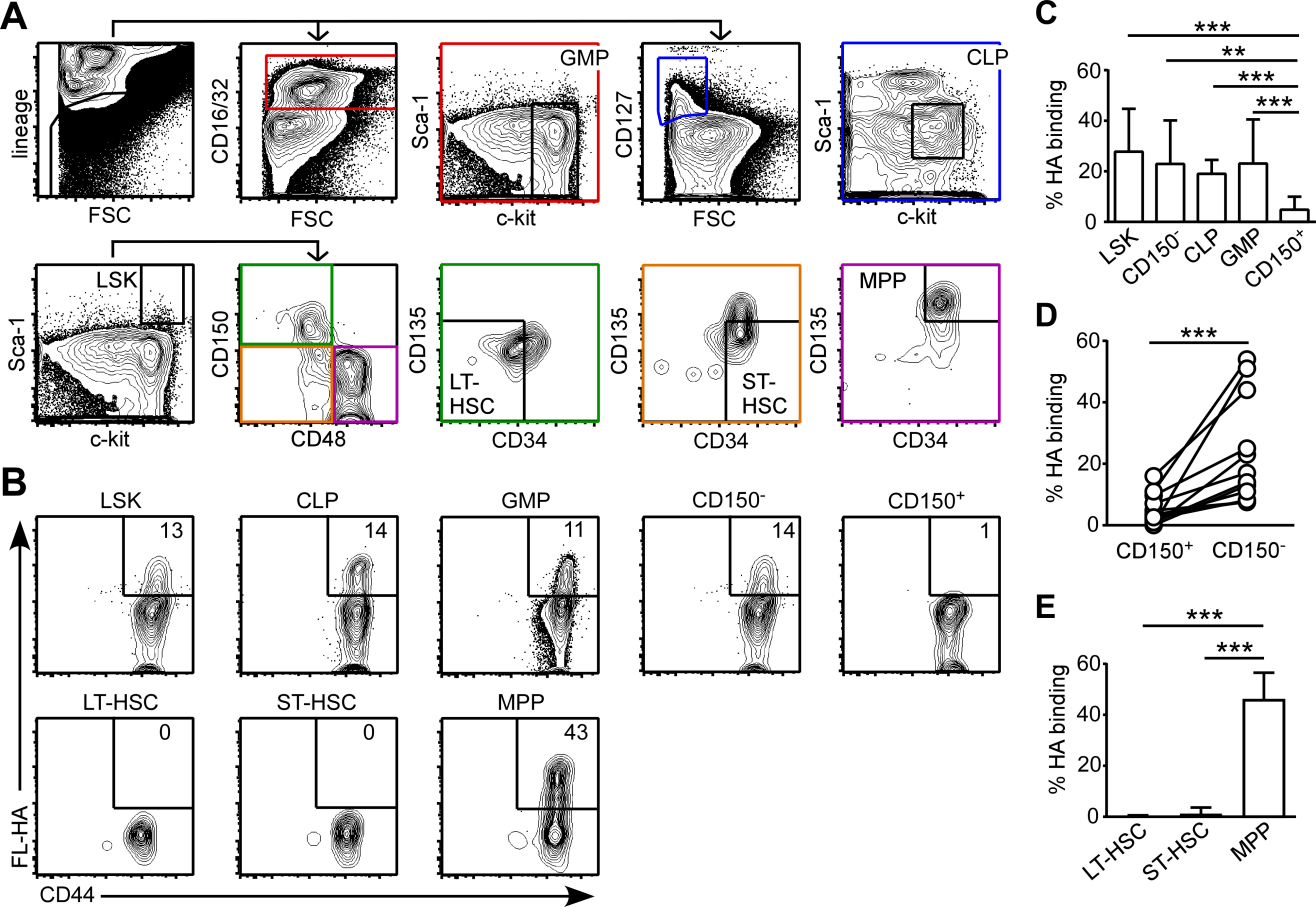
CD44 expression and HA binding by BM progenitors. (A) Gating strategies. (B) FC plots of FL-HA binding versus CD44 expression by BM LSK, CLP, GMP, CD150^+^ LSK, CD150^-^ LSK, LT-HSC, ST-HSC and MPP from CD44^+/+^ naïve mice. (C) Percentage of FL-HA binding by BM LSK, CLP, GMP, CD150^+^ LSK, and CD150^-^ LSK. (D) Percentage of FL-HA binding by CD150^+^ LSK and CD150^-^ LSK populations from the same mice as (C). (E) Percentage of FL-HA binding by BM LT-HSC, ST-HSC and MPP. Mean +/- SD from at least six biological replicates of two independent experiments. **p<0.01, ***p<0.001 calculated by Student’s t-test.

The ability for BMC with increased HA binding to better reconstitute the BM progenitors prompted the examination of CD44 expression and HA binding in these progenitor populations at steady state in CD44^+/+^ mice. Total, CD150^+^ and CD150^-^ LSK cells were identified as in Fig 6A. The common lymphoid progenitors (CLP) and granulocyte-monocyte progenitors (GMP) were identified based on expression of c-kit, Sca-1, CD127 and CD16/32 within in the lineage^-^ population in the BM (Fig 7A). The long- and short-term (LT and ST) HSC and MPP were identified based on their expression of CD150, CD48, CD34 and CD135 within the LSK population (Fig 7A). The LSK population showed high expression of CD44, yet only about 20% of the population bound FL-HA (Fig 7B and 7C). About 20% of CD150^-^ LSK, CLP and GMP populations bound FL-HA, whereas only about 7% of CD150^+^ LSK population bound FL-HA (Fig 7B and 7C). The percentage of FL-HA binding in the CD150^-^ LSK population was always higher than the percentage of FL-HA binding in the CD150^+^ LSK population in the same mouse (Fig 7D). Around 40% of MPP bound FL-HA, while little FL-HA binding was exhibited by LT- or ST-HSC (Fig 7B and 7E). At steady state, LT- and ST-HSC have a low turnover [31] compared to the MPP and other progenitors [32], raising the possibility that HA binding may be associated with their proliferation state.

### More HA binding BM LSK progenitors are in cell cycle

**Fig 8:**
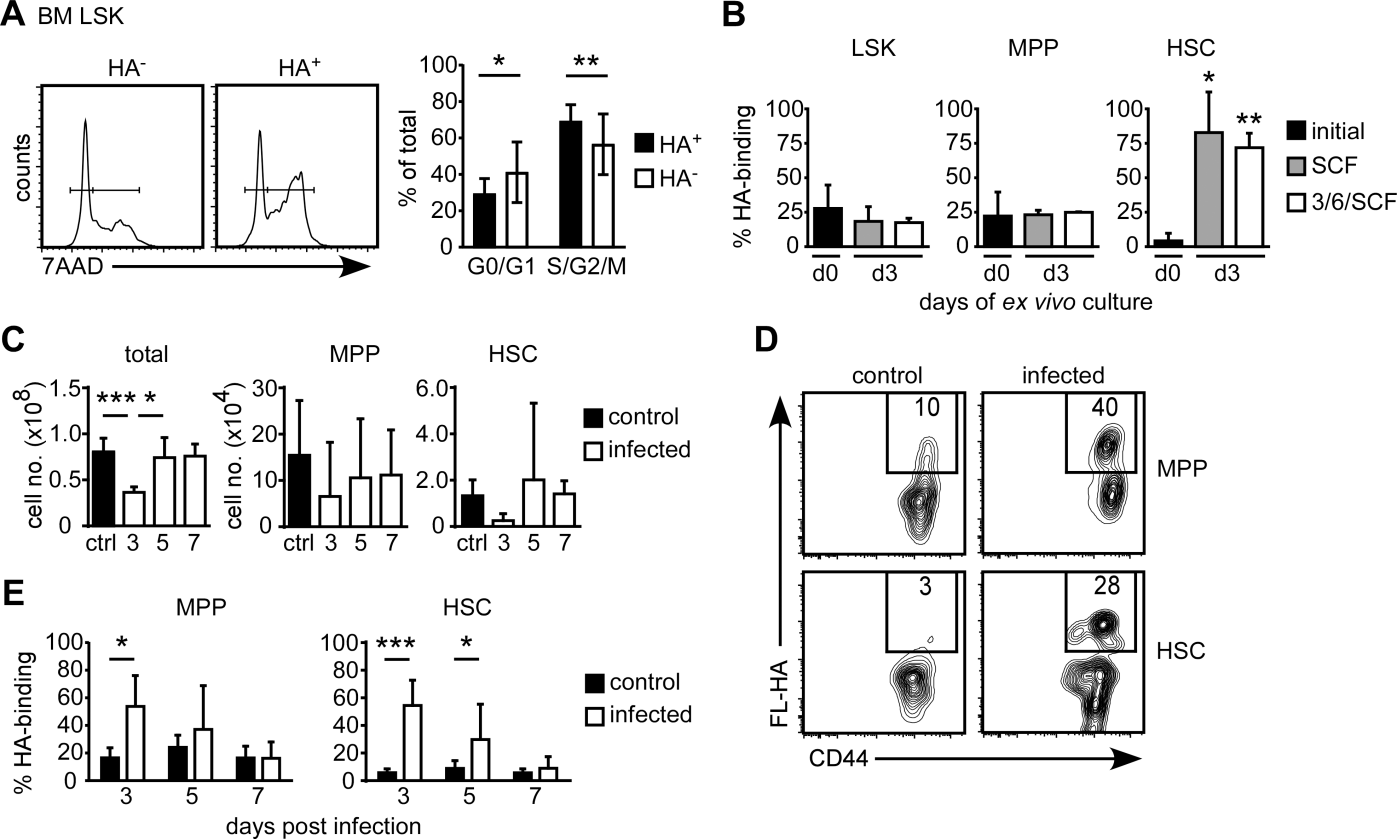
HA binding by BM progenitors is induced by proliferation. (A) Cell cycle analysis of BM LSK cells with 7AAD labeling. Histograms showing 7AAD labeling of HA-binding and non-binding BM LSK, and percentage of cells in G0/G1 and S/G2/M phases of the cell cycle averaged from nine mice over three experiments. (B) Percentage of FL-HA binding by total LSK, and MPP and HSC sub-populations before and after *ex vivo* culture in 100 ng/ mL SCF or in 3/6/SF medium for 3 days. (C-E) BMC were isolated from the femur and tibia of uninfected control mice, or mice that were i.v. infected with 30,000 CFU of *Lm* 3, 5 or 7 days prior. (C) Number of total, MPP, or HSC in the BM of the tibia and femur from both legs of each mouse. (D) FC plots of FL-HA binding versus CD44 expression by MPP and HSC in control and infected animals 3 days post infection. (E) Percentage of FL-HA binding by MPP and HSC in control and infected animals 3, 5 or 7 days post infection. Mean +/- SD from six biological replicates of two independent experiments. *p<0.05, **p<0.01, ***p<0.001 calculated by Student’s t-test.

To determine if HA binding was occurring on proliferating hematopoietic progenitor cells, BMC were labeled with 7AAD, to determine the stage of cell cycle. HA-binding and non-binding BM LSK cells were divided into G0/G1 or S/G2/M populations, and higher a percentage of HA-binding LSK were in the proliferative stages (S/G2/M) of the cell cycle than the non-binding LSK cells (Fig 8A). This shows that proliferating LSK cells are enriched in the HA-binding population.

### HSC increase their HA binding when induced to proliferate *in vitro*

To determine if HA binding was induced in proliferating hematopoietic progenitor cells, lineage-depleted BMC were cultured with either SCF, or a mixture of SCF, IL-3 and IL6, to induce their proliferation, and the percentage of cells that bound FL-HA were quantified 3 days later. While *ex vivo* cytokine stimulation had little effect on the percentage of HA binding cells within the LSK and MPP populations, the percentage of HA binding cells within in the CD150^+^ HSC was significantly increased from 7% to above 70% by culturing with SCF or the mixture of SCF, IL-3 and IL-6 (Fig 8B).

### MPP and HSC increase their HA binding *in vivo* in response to infection

To investigate if the proliferation of HSC and MPP increased HA binding *in vivo*, mice were infected with *Listeria monocytogenes* (*Lm*) i.v., as systemic bacterial infections induce proliferation of HSC and progenitor cells in the BM [33, 34]. Total BMC numbers were decreased on day 3 after infection, and then increased, returning to normal levels by day 5 (Fig 8C). Similar trends in cell numbers were also observed for the MPP and HSC, but were not significant, perhaps due to low cell numbers and high variability (Fig 8C). On day 3 post infection, a distinct FL-HA binding population was observed in the MPP and HSC (Fig 8D), and the percentage of FL-HA binding MPP and HSC was significantly higher in the infected animals 3 days post infection, where it increased from 18 to 50% for MPP and from 7 to 50% for HSC (Fig 8E). This increase remained significant at day 5 for HSC, and returned to control levels day 7 post-infection (Fig 8E). Thus the HA binding capacities of HSC and MPP are increased *in vivo*, in response to a systemic infection that induces their proliferation [33, 34].

### The addition of exogenous HA enhances hematopoietic progenitor cell proliferation

**Fig 9:**
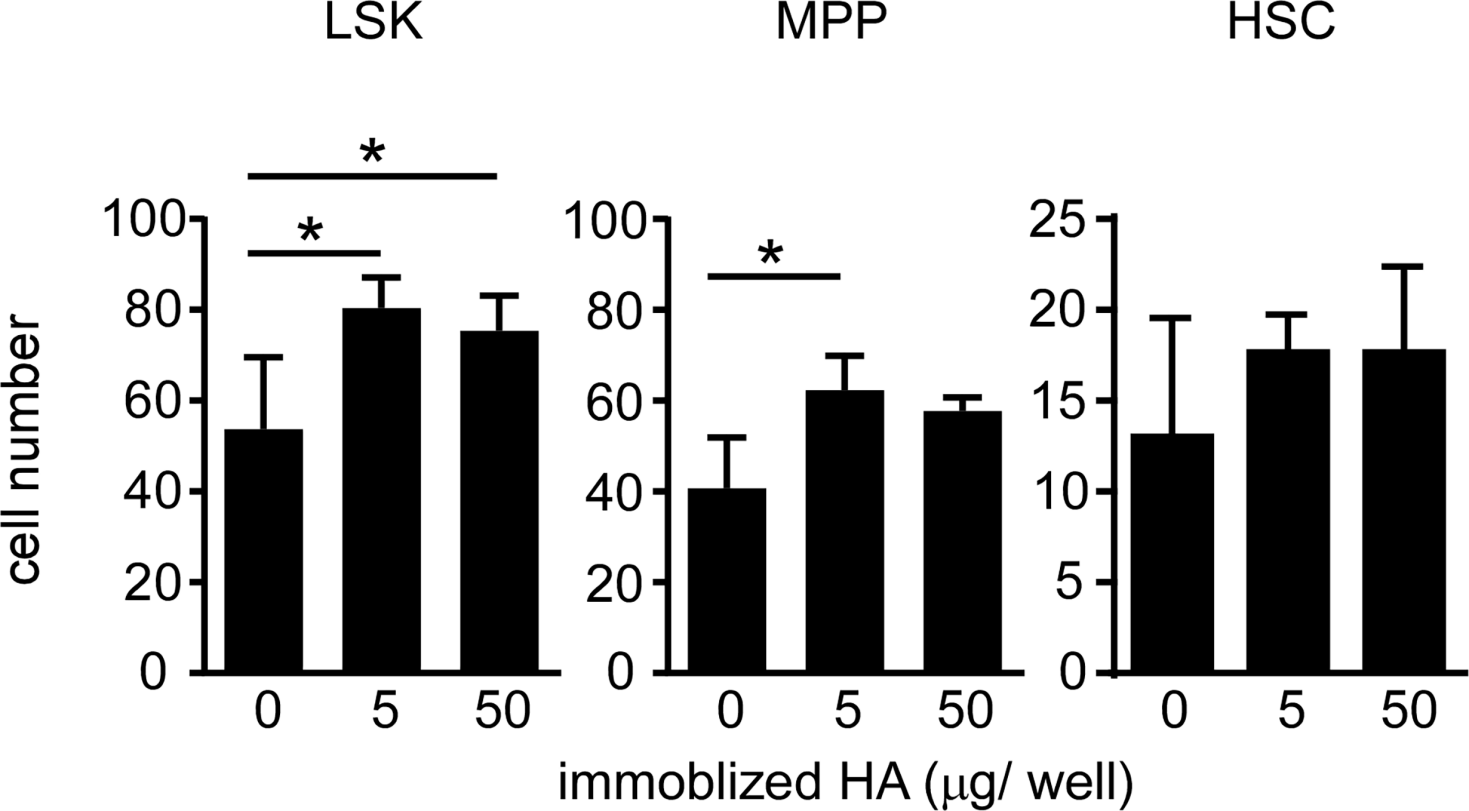
Exogenous HA enhances the proliferation of hematopoietic progenitor cells. 8 x10^4^ lineage-depleted BMC were cultured in 100 ng/ mL SCF with or without 5 or 50 **μ**g/ well of HA in a 96-well plate. Cells numbers were quantified 3 days later. Mean +/- SD from one representative experiment (out of two), each with three biological replicates. *p<0.05 calculated by Student’s t-test.

Having established that proliferating BM progenitors are enriched for HA binding cells, we wanted to determine if HA could promote their proliferation. Lineage-depleted BMC were isolated and cultured with SCF, in the presence or absence of exogenous, immobilized HA. After 3 days, LSK cells cultured with exogenous HA had significantly higher numbers of cells (Fig 9). Both MPP and HSC populations showed an increase, but significance was only reached in the MPP population, likely due to the low cell numbers of HSC. Thus HA can enhance hematopoietic progenitor cell proliferation.

## Discussion

Here, we identified a HA-binding population in eosinophils, NK and NKT cells at steady state. The use of LOF- and GOF-CD44 constructs to generate BMC expressing these mutants revealed a striking competitive advantage of HA-binding cells in the reconstitution of the hematopoietic system in irradiated recipients. This advantage occurred in the earliest hematopoietic progenitors, in the HSC and MPP within the LSK population. At steady state, HA-binding LSK cells were more likely to be in cell cycle and the percentage of HA-binding HSC was increased after proliferation *in vitro* and *in vivo* infection, and the addition of HA promoted the proliferation of hematopoietic progenitors *in vitro*.

A percentage of NK cells, NKT cells, eosinophils and the MPP population were capable of binding HA at steady-state. This was somewhat surprising given that few immune cell types bind FL-HA at steady state [1]. Typically, HA binding is induced upon activation of various immune cell types, including T, B, NK cells, macrophages, monocytes and monocyte-derived DC (reviewed in [1]). These activation signals also induce proliferation, and in activated T cells, the HA-binding T cells are the most actively proliferating [11], supporting a link between the induction of HA binding and the proliferative state of the cell. In this study, we also found a link between FL-HA binding and the proliferation of hematopoietic progenitors. HSC are normally quiescent and bound minimal FL-HA at homeostasis, whereas MPP, CLP and GMP actively turnover [32, 35, 36] and bound more FL-HA. Furthermore, factors that induce HSC proliferation and stimulate hematopoiesis, such as *in vitro* cytokine stimulation and *in vivo* systemic infection [33, 34], increased their FL-HA binding, and FL-HA binding cells were enriched for cells in cycle, further supporting FL-HA binding as a marker of proliferating BM progenitors. The addition of exogenous HA enhanced progenitor cell proliferation *in vitro*, suggesting a feed forward loop that may help sustain their proliferation.

In contrast to the competitive BM repopulation experiments with the GOF- and LOF-CD44 mutants, we did not observe a competitive BM progenitor advantage by naïve CD44^+/+^ BMC over CD44^-/-^ BMC, and no early hematopoietic progenitor defects were reported in CD44^-/-^ mice [21, 37]. One possible explanation for this is that in our gain and loss of function approach, we first cultured BMC in cytokines to induce the proliferation of hematopoietic cell progenitors to increase their transfection efficiency. As this proliferation also increases the HA binding potential of the CD44^+/+^ HSC compared to the HSC isolated from naïve mice, it may help distinguish the competitive differences between the HA-binding and non-binding populations.

Here, HA binding by BM progenitors provides a striking competitive advantage in BM engraftment, which could be due to an advantage in recruitment and/or localization to a proliferative niche within the BM. CD44 has been implicated in positively regulating human hematopoietic progenitor trafficking to the BM: a specific glycoform of CD44 in human hematopoietic progenitors mediates their migration to the BM [38]; incubation with an anti-CD44 monoclonal antibody, soluble HA or i.v. injection of hyaluronidase prior to transfer of human CD34+ blood cell into sub-lethally irradiated NOD-SCID mice reduces their homing [39]; and CD44 antibodies block the homing of human leukemic stem cells to mouse BM [40]. HA is present in the BM on the endosteum and sinusoids [39]. Its presence on the sinusoidal endothelium [39] could potentially enable HSC recruitment, which occurs in the sinusoids [41]. The sinusoids also provide a niche where the majority of HSC reside [39, 42–45], with a minority of quiescent HSC found in periarteriolar niches [31, 44] where HA is not expressed [39]. Proliferative HSC are more concentrated at the sinusoids [44], linking this HA-rich region with HSC proliferation. In our study, BMC engraftment preferentially occurred by the cells with the most HA-binding potential, which could augment recruitment and localization to the proliferative niche.

Previous studies have implicated CD44 and HA in HSC maintenance [46, 47]. Perivascular CXCL12 and SCF are important for the maintanence of hematopoeitic progenitors, and CXCL12 upregulates CD44-mediated HA binding [39]. Mice deficient in all three HA synthases have reduced HSC numbers, and their BMC have a reduced ability for reconstitution [46]. The injection of exogenous HA improved cell number recovery after 5-fluorouracil induced depletion [47], associating HA with increased cell numbers. We have shown that proliferating hematopoietic progenitors have increased HA-binding potential, which would promote their retention in an HA-rich sinusoidal niche. Retention to a niche rich in growth and survival factors [48], could provide an indirect effect of HA binding on survival and proliferation. However, we also found that exogenous HA directly promoted the proliferation of BM progenitors *in vitro*.

Murine LSK cells and human CD34^+^CD38^-^ BMC express low levels of HA independently of CD44, and the ligation of HA with an HA-binding protein on these cells potently inhibited cytokine-induced proliferation *in vitro* [49, 50]. In both immune cells and cancer cells, CD44 has been implicated in signaling via the PI3K/Akt pathway which promotes both proliferation and survival [51]. In HSC, Akt positively regulates cell cycling: double deletion of Akt1 and Akt2 reduces HSC long term reconstitution due to excessive quiescence [52], and constitutive pAkt signaling depletes HSC through excessive cycling [53]. CD44 ligation with an antibody induces PI3K/Akt signaling in tumor cells [54], and the interaction between CD44 and HA promotes the survival of tumor cells [55, 56] and alveolar macrophages [6]. Further, BM stromal cells that are incapable of synthesizing HA have a reduced ability to support HSC numbers and function [46]. Therefore, it is possible that HA binding is induced in hematopoietic progenitors during cell cycle and provides a direct signal to further promote their proliferation and/or survival.

Overall, we have shown that HA binding by BM progenitors is linked to the proliferative state of the cell, and their ability to bind HA confers a competitive advantage upon BM transfer into irradiated recipients. This opens up the possibility that modulation of HA binding by hematopoietic progenitors has potential clinical application to help improve BM engraftment and reconstitution in BM transplant recipients.
